# Annotations

**Published:** 1887-04-30

**Authors:** 


### Annotations.

"An ounce of practice is worth a pound of precept."

Ambulance and Self-help at Leeds.

The workmen of Leeds are going to show the workmen of London and other places the best and quickest way of securing an easy carriage for themselves in case of accident. They are not going to send round the hat, but to put their hands into their own pockets. The Leeds workers propose to celebrate the Queen's Jubilee by the free gift of a horse ambulance to the infirmary. The cost of the carriage will be from £80 to £100. Some people may say there is nothing very magnificent in this amount when it is remembered that the workmen who live in Leeds and the neighbourhood are numbered by scores of thousands, and that a farthing each from every operative would more than cover the whole outlay. That is no doubt true; still the step, though a short one, is in the right direction. Workmen have been so accustomed to think that hospitals and all their belongings should be provided by the better classes solely, that we are thankful to see them opening their eyes in the slightest degree to the duty of self-help. The rich should help the poor most certainly, and especially in times of sickness and accident; but we should greatly like to see workmen as a class rousing in themselves a feeling of manly independence, and taking their place among other classes as self-reliant and self-providing citizens. No country can be great except in the greatness of all its classes, and of the individual members of those classes.

Still Unreformed.

The election of an ophthalmic surgeon to the Cardiff Infirmary gave rise to no small excitement and difference of opinion, and evolved considerable feeling. It appears that at the hospital in question these elections are vested in the hands of the whole body of governors, so that the unfortunate candidate for any medical post has to spend large sums of money in an exhaustive canvass of the governors. In the old days a contested election for a medical post at any hospital gave rise in many places to as much, and even to more excitement, than a Parliamentary contest, and entailed an expenditure of energy and hard cash which few people nowadays can realise. The immediate cause of controversy at Cardiff the other day was the legality or illegality of 140 proxies, which had been procured by one of the candidates, but which were rejected by the chairman. Had these proxies been admitted, then the local candidate, Dr. Ensor, and not Mr. Thompson of Yorkshire, would have been elected by a substantial majority. We know nothing of the merits of this particular controversy, but we hope it may result in an alteration of the system of election at the Cardiff Infirmary. All the best managed hospitals appoint an election committee of 100 governors at each annual meeting, to which body is entrusted the duty of selecting a suitable officer for any vacancy which may occur on the medical staff during the current year. This system has answered admirably wherever it has been tried, and we can only hope that some similar reform will be instituted by the governors of this Welsh hospital without delay.

Grumblers.

There is no class of persons so worthy of the contempt of their fellows as the chronic grumbler. Malingering is a type only of this class of the *genus homo*, and we are not quite certain whether it is the worst type either. Who, for instance, can tolerate, or have patience with a man or woman who receives gratuitous, careful, kind, and efficient treatment in a hospital for several weeks, who leaves such an institution without complaint of any kind, and then turns round and declares that everything is bad, inefficient, and calculated to injure rather than to benefit the inmates? The latest example of this class of patient, which we believe to be rapidly disappearing, is recorded in the *North Wales Chronicle* of the 16th instant. A patient in the local cottage hospital is reported to have complained that the meals were given out for and served to the patients as they would be to a battalion of soldiers (a difficult matter, by the way, in a cottage hospital); that each patient received the same diet, whatever the state of his appetite or health; that the bread was served in hunks three-eighths to half an inch thick; that the presents sent to the patients were not all given to them, and sour rabbit, which had been cooked for several days, was served for dinner on at least one occasion. These charges exhibit a certain amount of ingenuity, combined with a careful elaboration of details. We are not surprised, however, that a special investigation by the Board of Management shows them to be unfounded. The sour rabbit, for instance, could not possibly have been sour, the nurses having partaken of it freely and found it perfectly good. The nurses invariably discharge their duties with care and attention, and the patients are well attended to in every respect. Such a finding upset the grumbler, "whose conduct was most disrespectful and his language coarse." The investigators conclude their report by stating that "if the grumbler's bearing whilst in the hospital was identical with that which he exhibited before the Committee of Investigation, the trouble and difficulty the nurses must have had with him whilst he was under their care can be more easily imagined than described" It is matter for congratulation that the grumbler belongs to a class of patient which at present tends to diminish rather than to increase in number.

### IN A CHEMIST'S SHOP IN DUBLIN.

(*Chemist weighing Calomel carefully.*)

*Customer:* "Arrah, thin! don't ye be so stingy, sorr! Shure! 'tis for a poor orphan child! Ye shouldn't be so mane!"

